# On the Constitutive Model of Nitrogen-Containing Austenitic Stainless Steel 316LN at Elevated Temperature

**DOI:** 10.1371/journal.pone.0102687

**Published:** 2014-11-06

**Authors:** Lei Zhang, Xiao Feng, Xin Wang, Changyong Liu

**Affiliations:** Key Laboratory for Advanced Materials Processing Technology of Ministry of Education, Tsinghua University, Beijing, China; Helmholtz-Zentrum Dresden-Rossendorf, Germany

## Abstract

The nitrogen-containing austenitic stainless steel 316LN has been chosen as the material for nuclear main-pipe, which is one of the key parts in 3rd generation nuclear power plants. In this research, a constitutive model of nitrogen-containing austenitic stainless steel is developed. The true stress-true strain curves obtained from isothermal hot compression tests over a wide range of temperatures (900–1250°C) and strain rates (10^−3^–10 s^−1^), were employed to study the dynamic deformational behavior of and recrystallization in 316LN steels. The constitutive model is developed through multiple linear regressions performed on the experimental data and based on an Arrhenius-type equation and Zener-Hollomon theory. The influence of strain was incorporated in the developed constitutive equation by considering the effect of strain on the various material constants. The reliability and accuracy of the model is verified through the comparison of predicted flow stress curves and experimental curves. Possible reasons for deviation are also discussed based on the characteristics of modeling process.

## Introduction

A nuclear main-pipe is a thick-walled steel tube that connects the reactor pressure vessel (RPV), steam generator, and reactor coolant pump. It is one of the seven first-class key parts of nuclear power plants [1]. Stress corrosion cracking (SCC) and Flow accelerated corrosion (FAC) of pipeline materials have resulted in several safety issues of nuclear plants in past decades. Thus, a material with good corrosion resistance should be used to build the main-pipe [2]. 316LN steel is a refined version of 316L steel. Compared with TP 316L, the strength and work hardening capability are improved as a result of the addition of nitrogen. Corrosion resistance is also improved as the carbon content is lowered. Furthermore, nitrogen additions enhance other qualities of the 316LN steel, such as resistance to pitting corrosion, crevice corrosion, and intercrystalline corrosion [3], [4].

A constitutive equation is a function that describes the relationship among several key parameters (such as stain, strain rate, temperature, etc.) during the deformation of a material. Without a reliable constitutive model, finite element analyses of hot working process will not produce accurate results. Hence, the constitutive equation is the foundation of steel hot-working research. The constitutive model is also frequently used as an input in FEM simulations to predict the material behavior under given loading and temperature conditions, such as those encountered during the hot-working process. There are mainly three types of metal constitutive equations: The first is the empirical equation, which often contains key parameters such as stain, strain rate, and temperature and is built up through mathematical modeling of experimental data, such as the Voce equation [5] or Johnson-Cook equation [6]. Constitutive equations of this kind are often mathematically well-built and can predict the change in flow stress caused by dynamic recovery. However, the accuracy of this kind of equations decreases as dynamic recrystallization takes place. The second type of constitutive equation is based on the theory of metal physics and can predict the flow stress change caused by work hardening and dynamic recovery with precision. Examples include the Zhou-Clode equation [7] and the Zerilli-Armstrong equation [8]. The third type of constitutive equation divides the flow stress curves into two components; the first can be either an empirical equation or an equation based on a physical theory, while the second introduces the softening mechanism, using the Avrami equation, to represent the effect of dynamic recrystallization, such as the Momeni-Abbasi equations [9].

Previous research on 316LN steels has mainly focused on how the corrosion resistance and fatigue performance are related to the composition and working technique [10]–[15]. Other researchers have investigated dynamic recrystallization in this steel after one or several hot-working passes in order to guide the present manufacturing technology of nuclear main-pipe [16]–[18]. However, most current constitutive models of 316LN steel do not take strain into consideration, and the development of a more accurate constitutive equation is very important.

The purpose of this study is to develop a constitutive model that can be used to predict the flow stress of 316LN steel during hot-working. This model takes into account of the effect of strain, strain rate, and temperature. In this research, isothermal hot compression tests were conducted over a wide range of strain rates (10^−3^–10 s^−1^) and deformation temperatures (900–1250°C). The reliability of the constitutive equation was assessed over the entire experimental temperature and strain rate range.

## Experimental Procedures

**Figure 1:**
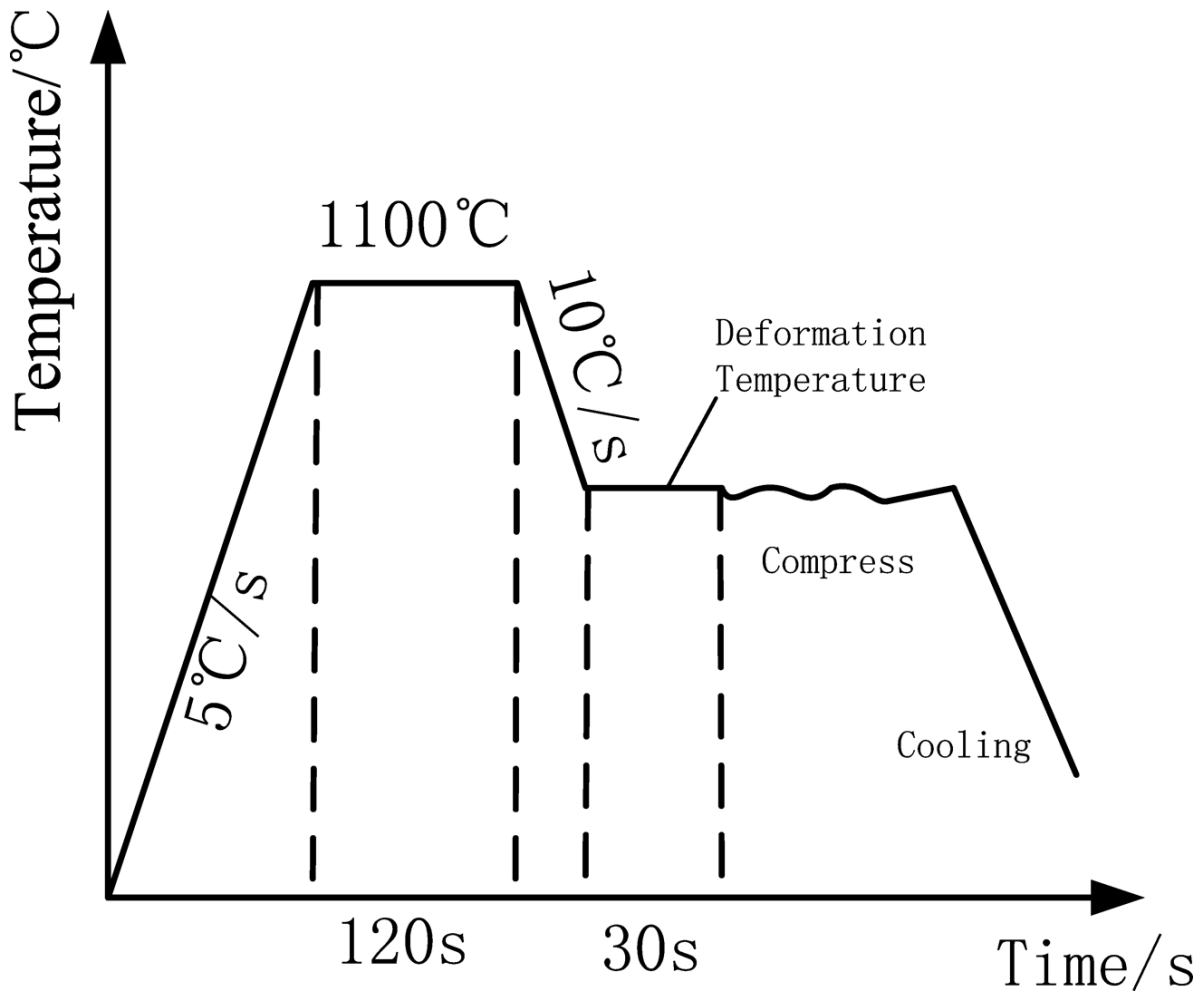
Heating profiles of the specimens before, during, and after the compression tests.

The chemical composition of the 316LN steel used in this research meets the US composition standard of TP 316LN, as shown in Table 1. The accuracy of the composition may seem to be disparate between different elements, this is because the method used for meassurement was different among different elements. For elements with very low content, methodswith very high accuracy were used, while for the elements with higher content, methods with lower accuracy were used. A Gleeble-1500D thermal simulation machine was used to measure the compression behavior at eight elevated temperatures (900, 950, 1000, 1050, 1100, 1150, 1200, 1250°C) and five different strain rates (10^−3^, 10^−2^, 10^−1^, 1, 10 s^−1^). Cylindrical specimens 10 mm in height and 6 mm in diameter were used. To reduce the effect of friction, both tantalum and graphite sheets were used as a lubricant. The true strain at the end of the compression test is fixed at 0.693. The heating profile is illustrated in Fig. 1. Prior to the compression test, each specimen was heated to 1100°C at a rate of 5°C/s and held for 2 minutes to insure the specimens were fully austenized. Then, the temperature was dropped to one of the experimental temperatures listed at a rate of 10°C/s and held for 30 seconds.

**Table 1:** Chemical composition of the 316LN steel used in this study (wt.%).

C	Si	Mn	P	S	Cr	Ni	Mo	N
0.03	0.56	1.05	0.031	0.0042	16.47	10.75	2.11	0.12

## Results and Discussion

### 3.1 Experimental results

**Figure 2:**
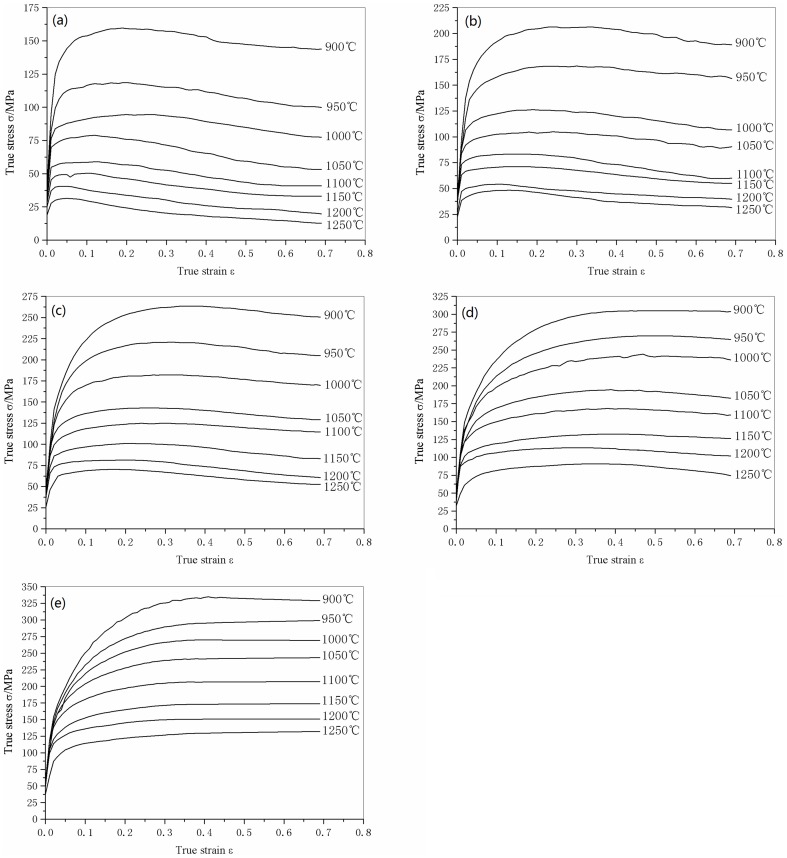
Flow stress curves of 316LN steels compressed at different temperatures and strain rates. (a) 10^−3^ s^−1^, (b) 10^−2^ s^−1^, (b) 10^−1^ s^−1^, (b) 1 s^−1^ and (b) 10 s^−1^.

The true stress-true strain curves obtained from the experiments are shown in Fig. 2. It is clear that the flow stresses of 316LN steel are strongly affected by the experimental temperature, strain rate, and level of strain. Higher levels of flow stress are more likely at lower temperatures and higher strain rates. In addition, dynamic recrystallization is also affected by temperature and strain rate. The peaks on the flow stress curves, which indicate that dynamic recrystallization took place, can be observed only at relatively high temperatures (1000–1250°C) and low strain rates (10^−3^–10^−1^ s^−1^). At the other conditions, there are no obvious peaks on the flow stress curves, which means that the material softens by dynamic recovery rather than dynamic recrystallization. This is in good agreement with previous research [19].

### 3.2 Constitutive equation

The flow stress is mainly affected by deformation temperature, strain rate, and strain under hot deformation conditions. To simplify the analysis, the effect of temperature and strain rate were examined first. The Arrhenius type equation is typically used to describe the correlation between strain rate, deformation temperature, and flow stress, particularly at high temperature, and the effects of the temperature and strain rate on the deformation behavior can be characterized by the Zener-Hollomon parameter (Z) in an exponential equation [20]. These two equations are
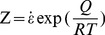
(1)and
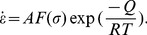
(2)
*F*(*σ*) is a function that has different forms at different stress levels:
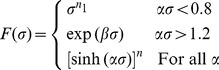
(3)In the above, *Q* is the activation energy of hot deformation (in J/mol), *R* is the universal gas constant (8.314 J/mol^−1^K^−1^), *T* is the absolute temperature (in K), *σ* is the stress (in MPa), and 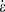 is the strain rate (in s^−1^). *A*, *α*, *β*, *n*, and *n*_1_ are parameters that depend on the particular features of the material. *α* and *β* are related by *α* = *β*/*n*_1_.

To build the constitutive model for 316LN steels, the material constants need to be determined. The true stress-true strain data obtained from the compression tests can be used to evaluate these constants. In this research, a deformation strain of 0.2 is taken as a key point to demonstrate the solution procedure for the material constants. At a certain temperature, for the low stress level (*ασ*<0.8) and high stress level (*ασ*>1.2) behavior, substituting the power law and exponential law of *F*(*σ*) into Eq. (2), respectively, the following equations can be obtained:
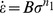
(4)and
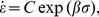
(5)in which *B* and *C* are both material constants that are not affected by the deformation temperature. Taking the logarithm of both sides of Eq. (4) and Eq. (5), the following equations can be obtained:
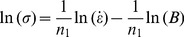
(6)and
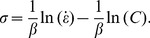
(7)


**Figure 3:**
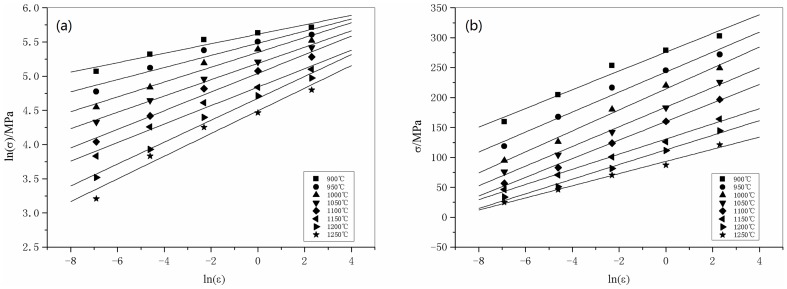
Linear regression fits of (a) ln *σ*-ln 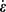 and (b) *σ*-ln 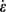 data. (All data involved.)

The plots of ln *σ*-ln 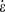 and *σ*-ln 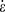 are shown in Fig. 3, and these data have been fit with lines according to Eqs. (6) and (7), from which the slopes are tantamount to 1/*n*_1_ and 1/*β*, respectively. The determination of *α*, *β*, *n*_1_ is performed in two steps. As shown in Fig. 3, the slopes of the ln *σ*-ln 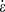 and *σ*-ln 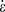 plots vary with temperature. In step one, the average value of the slopes is used when determining the estimated value of *n*_1_ and *β*. The estimated values of *n*_1_ and *β* are 8.1338 and 0.0684 MPa^−1^, respectively. Hence, the estimated value of *α* = *β*/*n*_1_ = 0.008414 MPa^−1^.

**Figure 4:**
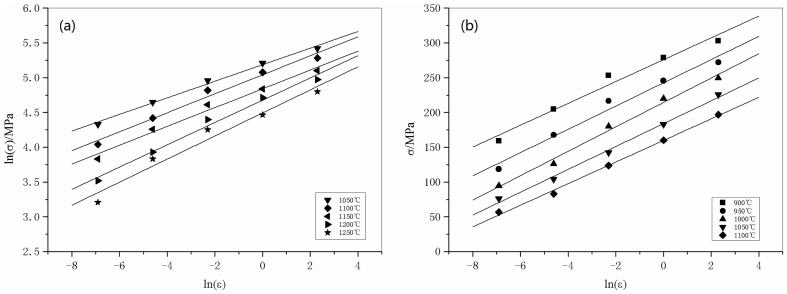
Linear regression fits of (a) ln *σ*-ln 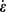 and (b) *σ*-ln 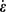 data. (Selected data.)

The values of *α*, *n*_1_, and *β* acquired by this method are not the exact values, because Eqs. (6) and (7) are intended for low and high levels of stress, respectively. In the previous solution procedure, the effect of stress level has not been considered. Therefore, in step two, to determine the accurate values of *α*, *n*_1_, and *β*, the values of *ασ* are examined to determine whether each flow stress curve corresponds to a high or low stress level. Then, a linear regression procedure is applied to the selected key points again to determine corrected values of *α*, *n*_1_, and *β*. For instance, it is found that for the stress-strain curves collected at relatively low temperatures, e.g. 900–1000°C, *ασ*>1.2, so these curves were treated as high stress level data. Accordingly, Eq. (6) no longer suits the situation. Data points from these curves should be excluded while carrying out the linear regression procedure based on Eq. (6). For temperatures in the range of 1150–1250°C, *ασ*<0.8, i.e., the stress levels are low, so data points from these curves should be excluded while carrying out the linear regression procedure based on Eq. (7). As Fig. 4 illustrates, when the data points from the improper curves are excluded from the regression analysis, the results are *n*_1_ = 6.977, *β* = 0.0611 MPa^−1^, *α* = 0.008757 MPa^−1^.

The hyperbolic sine forms of *F*(*σ*) are suitable for both high and low level stresses, and substituting the hyperbolic sine form of *F*(*σ*) into Eq. (2), Eq. (8) can be obtained:
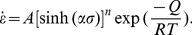
(8)


Taking the logarithm of both sides produces:
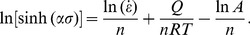
(9)


**Figure 5:**
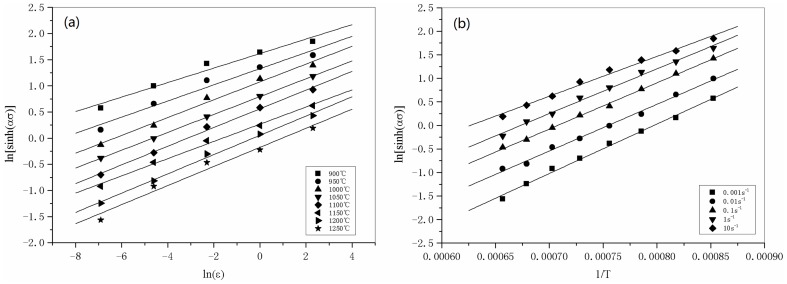
Linear regression fits of (a) ln (sinh *ασ*)-ln 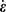 and (b) ln (sinh *ασ*)-1/*T*.



By substituting the values of the flow stress and corresponding strain rate at a strain of 0.2 for all the deformation temperatures into Eq. (9), the relationship between ln (sinh *ασ*) and –ln 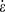 for a particular temperature can be obtained. These curves are shown in Fig. 5a. The value of *n* can be easily derived as the average of regression line slopes, giving *n* = 5.9029. For a particular strain rate, differentiating Eq. (9) leads to

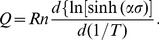
(10)Therefore, the activation energy of hot deformation of 316LN can be calculated as the slope of the ln (sinh *ασ*) vs. 1/*T* regression line. From Fig. 5, the value of *Q* can be evaluated as 477.32 kJ/mol by averaging the values of *Q* obtained at different strain rates. By substituting all the known parameters into Eq. (9), one can determine the value of the material constant *A* (at a strain of 0.2) from the intercept of ln (sinh *ασ*) vs. ln 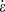. This gives ln *A* = 38.83.

Previous analysis determined the material constants (*n*, *Q*, *α*, ln *A*) at a certain strain (*ε* = 0.2), thus a constitutive model without considering the effect of strain was constructed. However, the value of the flow stress at elevated temperatures is strongly affected by the strain, especially in the initial stages of deformation, as can be seen in Fig. 2. Therefore, to build up a constitutive model with good accuracy, the effect of strain is needs to be considered.

### 3.3 Compensation for strain

**Figure 6:**
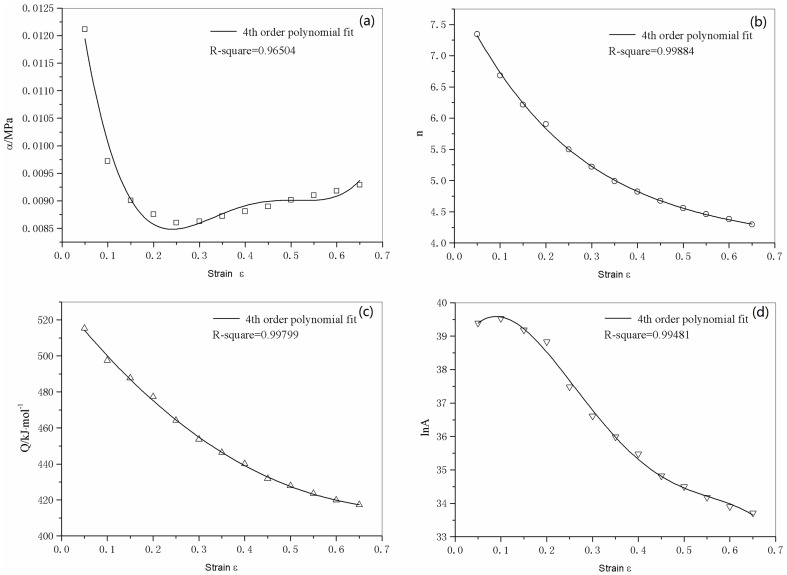
4th order polynomial fit of the material constants. (a) *α*, (b) *n*, (c) *Q*, and (d) ln *A*.

The effect of strain is incorporated by assuming that the strain affects the values of material constants *n*, *Q*, *α*, and ln *A*. Over the range of strain 0.05–0.65, a strain-stress data point was picked out every 0.05. The value of material constants were calculated at these different strains, then several polynomial fits were carried out to determine the relationship between strain and material constants. The result of these polynomial fits are shown in Fig. 6.

To determine the proper order for this polynomial, the order of polynomial fit was taken as 3, 4, and 5. A 4^th^-order polynomial, as shown in Eq.(11), was found to be both accurate and simple. In fact, as will be stated later, the expeced error for 3rd and 5th polynomial fit were larger than 4th-order fit. The coefficients of the polynomial fits for each constant are given in Table 2.

**Table 2:** Coefficients of the polynomial fit for *n*, *Q*, *α*, and ln *A*.

*α*	*n*	*Q*	ln *A*
B_0_ = 0.01498	C_0_ = 8.027	D_0_ = 529.7547	E_0_ = 38.40514
B_1_ = −0.07450	C_1_ = −15.39	D_1_ = −321.312	E_1_ = 29.44497
B_2_ = 0.3002	C_2_ = 26.43	D_2_ = 246.6598	E_2_ = −215.3186
B_3_ = −0.4970	C_3_ = −23.29	D_3_ = −19.21761	E_3_ = 404.8296
B_4_ = 0.2942	C_4_ = 8.467	D_4_ = −13.33608	E_4_ = −247.0318



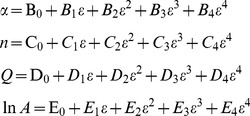
(11)


By substituting Eq.(1) into Eq.(8), the constitutive model of 316LN steel can be expressed as a function relating the flow stress to the Zener-Hollomon parameter and material constants. This gives
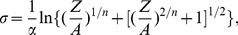
(12)where the material constants are taken as given in Eq.(11).

**Figure 7:**
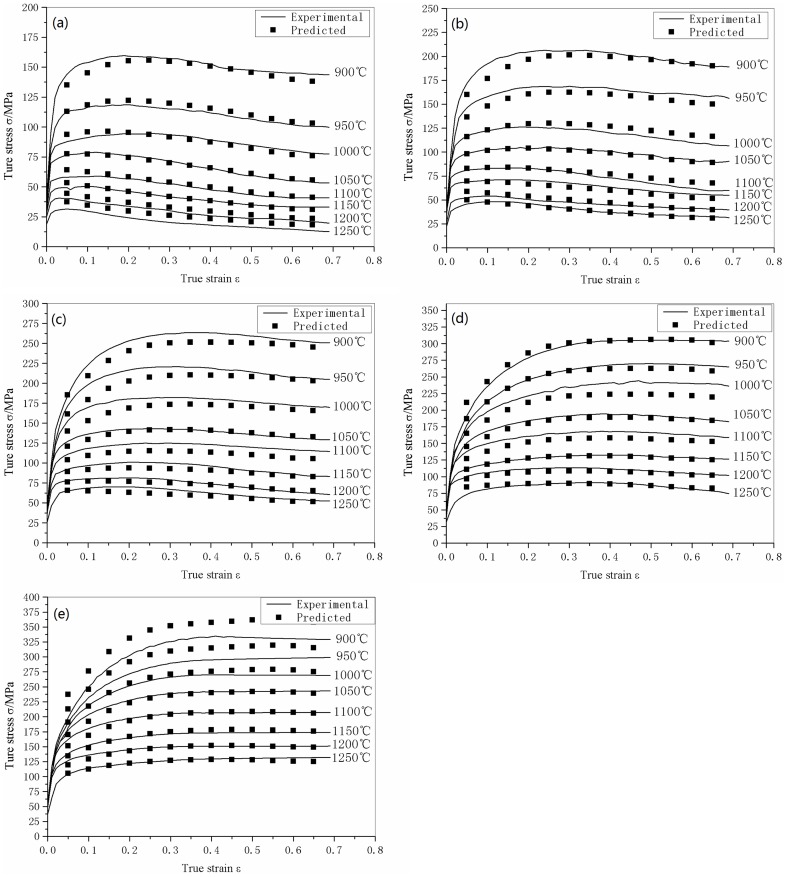
Comparison of experimental curves and predicted curves at various strain rates. (a) 10^−3^ s^−1^, (b) 10^−2^ s^−1^, (b) 10^−1^ s^−1^, (b) 1 s^−1^ and (b) 10 s^−1^.

The flow stress curves can be obtained from the constitutive model. A comparison of the predicted flow stress curves and the experimental curves was conducted in order to verify the accuracy of the model. The result of the comparison is in Fig. 7; it can be seen that the predicted results are in good accordance with the experimental results. The largest deviation occurs at 900°C and a strain rate of 10 s^−1^. The comparison between the predicted flow stress values and the experimental ones are shown in Table 3, the average relative error was calculated using:

**Table 3:** The relative error between the predicted curve and experimental curve at 900°C and 10 s^−1^ and the given strain.

*ε*	σ_e_ (MPa)	σ_p_ (MPa)	Error (%)
0.05	198.585	237.3224	19.50671
0.1	250.614	276.10674	10.17211
0.15	282.848	308.57054	9.09412
0.2	302.683	331.26437	9.44267
0.25	317.821	344.63529	8.43692
0.3	325.581	351.55219	7.97688
0.35	331.126	355.07918	7.23386
0.4	333.998	357.36067	6.99485
0.45	333.781	359.4846	7.70074
0.5	332.375	361.63752	8.80407
0.55	331.389	363.19427	9.59756
0.6	330.491	362.74229	9.75860
0.65	329.667	358.30444	8.68678
Average	-	-	9.49



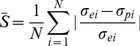
(13)where *σ_ei_* and *σ_pi_* are the experimental value and predicted value of flow stress, respectively. *N* is the total number of data points involved.

The average relative error for stress at 900°C and 10 s^−1^ is 9.49%. For for 3rd and 5th polynomial fit, the expected errors were 10.31% and 10.19%, respectively. While experimental errors during hot compression and the theoretical uncertainties in the physical models may cause the deviation, the modeling procedures also effect the result. As stated in section 3.2, when determining the material constants *n*_1_ and *α*, according to the application ranges of the power-law and exponential equations, the high stress level (*ασ*>1.2) curves and low stress level (*ασ*<0.8) curves were excluded respectively. Thus, the value of *n*_1_ derived through linear regression may not be so accurate for the low stress level conditions, similarly, the value of *α* may not be so accurate for the high stress level conditions. Therefore, in high stress level condition (900°C and 10 s^−1^), the deviations are larger than other conditions.

Generally speaking, acceptable agreement was achieved between predicted results through the proposed constitutive equations and experimental data, which indicates the constitutive model established in this research is reliable and with good accuracy.

## Conclusion

In this study, the flow stress curves of 316LN steel at elevated temperature and various strain rates are obtained through isothermal hot compression tests. A constitutive model of 316LN steel is also developed based on the experimental data. The following conclusion can be drawn:

1. The flow stress of 316LN steel at elevated temperature is strongly affected by deformation temperature, strain rate, and strain level, and the value of the flow stress increases with lower deformation temperatures and higher strain rates.
2. The four material constants (*n*, *Q*, *α*, and ln *A*) are all relative to strain. For instance, the hot deformation activation energy (*Q*) decreases with increases in strain. The average hot deformation activation energy at different strain rates is 477.32 kJ/mol at a strain of 0.2.
3. The constitutive model developed in this research is reliable, with the deviation of 9.49% in the worst case (900 °C and 10 s^−1^) and a deviation below 5% in most other cases. The good accuracy of the model makes it reliable to be applied to guide the hot working of TP 316LN steel.
